# Early-life family income and subjective well-being in adolescents

**DOI:** 10.1371/journal.pone.0179380

**Published:** 2017-07-17

**Authors:** Genevieve Gariepy, Frank J. Elgar, Mariane Sentenac, Christopher Barrington-Leigh

**Affiliations:** 1 Institute for Health and Social Policy, McGill University, Montreal, QC, Canada; 2 Department of Psychiatry, McGill University, Montreal, QC, Canada; 3 Department of Economics, McGill University, Montreal, QC, Canada; Queensland University of Technology, AUSTRALIA

## Abstract

**Purpose:**

Subjective well-being (SWB) in youths positively relates to family income, however its association with income during childhood is unclear. Using longitudinal data from the US Panel Study of Income Dynamics (n = 2234 adolescents, age 12–19 years), we examined whether the timing and duration of low family income in childhood was associated with adolescent SWB.

**Methods:**

We categorized family income during childhood into state-specific quintiles. Adolescent SWB was assessed using a 12-item questionnaire (score range 3–18). We used marginal structural modelling to test for sensitive periods of exposure to low income and tested cumulative effects of income by modelling the number of years spent in the poorest income quintiles.

**Results:**

A period in early childhood (age 0–2 years) was particularly sensitive to low family income. Adolescent SWB was 1.65 (95% CI 0.40, 2.91) points lower in those who grew up in the poorest income quintiles during early childhood compared with the top quintile. Further, each childhood year spent in the poorest income quintiles was associated with a 0.10 point (95% CI 0.04, 0.16) lower SWB score in adolescence.

**Conclusions:**

The timing and duration of low family income in childhood both predict individual differences in adolescent SWB. Further studies are needed to clarify the mechanisms of these models and inform public policies.

## Introduction

Adolescence is a formative phase of human development, marked by significant physical, cognitive and social changes. Adolescents who report greater subjective well-being (SWB) fare better at schools, have better relationships, and have less mental health problems [1–3], and these benefits carry into adulthood [4–6]. SWB is a broad term that refers to a family of positive mental health measures which attempt to capture the way individuals evaluate their lives, including positive emotions, satisfaction, and meaning [7]. Positive psychology underlines the importance of both hedonia (“feeling good”) and eudaimonia (“doing well”) in determining SWB, such that SWB encompasses positive emotions (hedonic well-being) as well as meaning, purpose, engagement, and positive relationships (eudaimonic well-being) [3, 8–12]. Recent qualitative research found that adolescents endorsed SWB as involving multiple aspects of life, such as having good relationships and having life goals [13]. Despite the many benefits of SWB, little is known about the contextual factors that support or hinder SWB in adolescence.

Growing up economically disadvantaged might affect the development of SWB in youth. A large body of work has linked poverty and socioeconomic factors to poor mental health outcomes in youth [14, 15]. Children living in lower income families tend to experience more interpersonal violence, financial strain, family turmoil, and environmental hazards and are at increased risk of injury, engage in more health compromising behaviours, and exhibit more social skills deficits and behavioral problems [16, 17]. They have less access to material goods that have become social norms [18] and may feel a sense of deprivation compared to others [19]. Further, living in a family at the bottom of the income distribution might affect parental well-being, influencing their parenting skills and ultimately the well-being of their child [18, 20]. Quasi-experimental studies found beneficial effects of reducing poverty on mental health problems in youth [21, 22]. To further understand the elements that make it possible for adolescents to lead mentally healthy lives, research efforts that focus on positive mental health, in addition to mental health problems, are needed.

Current knowledge on the link between socioeconomic factors and SWB in adolescents is sparse and limited to cross-sectional evidence [23–26]. Longitudinal data with multiple follow-up assessments are needed to track socioeconomic exposures throughout childhood and their influence on SWB in adolescence. The sensitive period model of life course research posits that there are sensitive stages during childhood when family income has a lasting impact on the development of SWB [27]. For example, evidence from neuroscience and developmental psychology indicates that the first years of life are crucial for brain development and may be a time when children are particularly sensitive to their environment [28]. The accumulation of risk model suggests that the cumulative effect of living in a low income family throughout childhood affects SWB in adolescents [27]. The cumulative cost of managing stress over time may be what wears on the body and brain of children and affects mental health. The compounded effects of low income in childhood on the psychological, social and cognitive development might also affect well-being.

In this study, we examined the association between family income during childhood and adolescent SWB, and tested the sensitive period and accumulation of risk models as potential mechanisms. We further conducted analyses by specific domains of SWB (emotional, social, and psychological well-being). We hypothesized that the consequences of family income in childhood on adolescent SWB were shaped by the duration and timing of the exposure.

## Methods

### Study population

The PSID is a nationally representative longitudinal household survey of nearly 5000 American families followed annually from 1968–1996 and biennially from 1997–2007 [29]. A sub-cohort of immigrant families was added in 1997–1999. In 1997, the PSID initiated the CDS to collect information on children of the cohort. Three waves of CDS data are available: CDS-I (1997, 88% response rate), CDS-II (2002–03, 91% response rate) and CDS-III (2007–2008, 90% response rate). The CDS-II and CDS-III asked adolescents aged 12 to 19 a set of questions related to SWB [30]. This study included participants of the CDS II and III with data on SWB (n = 2234). Adolescents in our sample were between 12 and 19 years old and were born between 1984 and 1996. We merged CDS adolescent data with PSID parental data. The PSID measures family characteristics and income every year (1968–1996) or two years (1997–2007) of the child's life from birth to adolescence. The PSID was approved by the University of Michigan Institutional Review Board and secondary analysis of the PSID data was approved by the McGill University Institutional Review Board.

### Measures

#### Subjective well-being

The CDS-II and III asked adolescents to rate how often in the past month they experienced 12 indicators of positive mental health related to three domains: emotional (3 items, alpha = 0.84), social (5 items, alpha = 0.80) and psychological (4 items, alpha = 0.78) well-being (6). Responses were on a 6-point scale ranging from never (1) to every day (6). The items were adapted from MacArthur MIDUS Youth and work by Keyes [31, 32]. Details about the items and response scale are presented in S1 Table. The mean response score for each domain was calculated (range 1–6) and the total SWB score was computed by summing the scores across the three domains (range 3–18) [3]. SWB was measured at one time point during adolescence (ages 12–19 years old). For the limited number of respondents that took part in both waves II and III of the CDS during their adolescence (n = 590), we used SWB score from wave II in the analyses. In sensitivity analyses, results were unchanged when using scores from wave III.

#### Family income

We obtained total family income after taxes and government transfers from the PSID-Cross-National Equivalent File [33]. We categorized family income into quintiles using thresholds estimates of household income quintiles by state and year from the Integrated Public Use Microdata Series (https://cps.ipums.org/cps/index.shtml). Quintiles provide more stable estimates with our statistical approach [34] and facilitate the interpretation of sensitive periods and cumulative effects associated with low family income. In sensitivity analyses, we used the continuous amount of family income in 2013 USD, which was transformed using an inverse hyperbolic sine function, to account for the standard diminishing returns to income.

#### Covariates

Based on our literature review, we adjusted for parental variables that could confound the relationship between family income and adolescent SWB. Parental data were collected at each PSID survey waves dating back to the birth of the child. The variables included time-invariant variables, including sex, race or ethnicity (white; black; other) and age of the primary caregiver when the child was born, and year of birth; and time-varying variables, including years of education, marital status (married or living with partner; other) and working status (working full-time; part-time; not working) of the primary caregiver; number of people and number of children in the family; state of residency; and state median income. Primary caregivers were primarily mothers (97%). Historical data on state median household income in 2013 USD were obtained from the US census bureau (http://www.census.gov/hhes/www/income/data/historical/household/).

### Statistical methods

#### Sensitive period model

We divided childhood data into four periods, representing development stages based on the US Centers for Disease Control and Prevention [35]: early childhood (0–2 years old); preschool years (3–5 years old); middle childhood (6–8 years old); and pre-adolescence (9–11 years old). We assessed the sensitive period model using marginal structural modeling (MSM), a relatively novel method that takes endogenous confounding into account (see S1 Fig for diagram) [36, 37]. MSM uses inverse probability weights (IPW) to create a weighted sample in which exposure to a family income quintile is not confounded by measured covariates (see S1 File for model specifications and details). Extreme IPW values below the 1^st^ or above the 99^th^ percentile were replaced with the value of the respective percentile (n = 36). We ran a linear regression using IPW to estimate the joint effects of family income quintiles across all childhood periods on SWB at adolescence.

#### Accumulation of risk model

We modelled the number of years spent in the two poorest income quintiles during childhood and tested its association with adolescent SWB in a linear regression, adjusted for time-invariant and time-varying covariates at baseline. The income threshold for the two poorest quintiles corresponds to about twice the federal poverty level for a family with two children [38]. Research indicates that an income of about twice the federal poverty threshold is what an average family needs just to afford basic expenses. In sensitivity analyses, we checked if results were similar when modelling the number of years spent in the poorest income quintile only. We imputed missing values on family income quintile using multiple imputation by chained equations. We used fractional polynomial regression to check for non-linearity and determined that a linear model fit the data best.

#### Sensitivity analyses

We conducted several sensitivity analyses: 1) we conducted stratified analyses by SWB measured in early (12–15 years old) and late adolescence (16–19 years old) because we found that SWB was inversely related to age during adolescence. 2) Participants with missing data were similar to those without (S2 Table), except that they were more likely to be part of the late panel entry of immigrant families in 1997–1999, from larger families, and of race or ethnicity other than black or white. We performed multiple imputation using the *mi impute* chained procedure and pooled the results across five imputed data sets using *mi estimate* procedures in Stata. 3) In the accumulation of risk model, preliminary analyses revealed a higher probability of living in the two poorest income quintiles during the first three years of childhood. We ran the model excluding the first three years of childhood and 4) ran the model adjusting for family income quintile during the first three years. 5) The distribution of years spent in the two poorest income quintiles was highly skewed and we worried extreme values might affect associations. We therefore restricted analysis only to those who lived in the two poorest income quintiles for 5 years or less. 6) We tested whether cumulative exposure to low family income affected adolescents independent of their current family income quintile by adjusting for current family income quintile in the model. 7) We additionally checked whether changes in family characteristics between early childhood and adolescents might impact results by including family characteristics in both early childhood and adolescence in the same model.

All analyses incorporated child-level survey weights provided by PSID to adjust for sample selection and attrition. Robust standard errors were computed in all models. Statistical analyses were performed in STATA (version 12.1, Stata Corp, College Station, TX).

## Results

Table 1 shows characteristics of the sample at baseline (year of birth). The sample included 1108 boys (50.3%) and 1093 girls (49.7%). Caregivers were 29 years old on average (SD 6.5) and the majority were married (78.7%), white (72.8%), had at least a high school education (89.7%), and worked either part-time (46.3%) or full-time (21.3%). The mean adolescent SWB score was 12.9 (SD 2.8). Adolescents were on average 14.1 (SD 1.7) years old when they were asked about their SWB (age range 12–19 years old).

**Table 1:** Baseline characteristics of adolescents with information on subjective well-being in the child development supplement of the panel study of income dynamics, United States, 2002 and 2007 (n = 2234).

	Weighted % (n)	Weighted mean (SD)
**Characteristics of Primary Caregiver**		
Age (years)		29.0 (6.5)
Sex		
Women	96.7 (2159)	
Men	3.4 (75)	
Race/ethnicity		
White	72.8 (1137)	
Black	16.2 (945)	
Other	11.0 (152)	
Marital status		
Married/partnered	78.7 (1291)	
Not married/partnered	21.3 (559)	
Years of education		13.4 (2.1)
Working status		
Full-time	21.1 (413)	
Part-tine	46.3 (791)	
Not working	32.6 (649)	
**Characteristics of Family**		
Number of family members		4.0 (1.2)
Number of children in the family		2.0 (1.1)
State median household income (in 2013 USD)		51 128 (7613)
**Characteristics of Participants**		
Year of birth		
1984	0.6 (23)	
1985	8.5 (173)	
1986	7.6 (182)	
1987	8.9 (226)	
1988	9.2 (189)	
1989	8.5 (178)	
1990	9.1 (196)	
1991	10.3 (223)	
1992	7.6 (169)	
1993	10.1(216)	
1994	12.6 (313)	
1995	6.9 (143)	
1996	0.1 (3)	

n: sample size; SD: standard deviation; USD: United States dollars

### Sensitive period model

Table 2 presents the results of the MSM to test for sensitive periods. Family income quintile during early childhood had a direct effect on adolescence SWB. Growing up in the first, second and third poorest quintile groups in early childhood was associated with an adolescent SWB score that was 1.65 (95% CI: 0.40, 2.91), 0.63 (95% CI: -0.32, 1.58) and 0.82 (95% CI: -0.10, 1.74) points lower, respectively, compared to the richest quintile group, although confidence intervals for the second and third quintiles crossed the null. Results remained robust to sensitivity analyses (S3 Table).

**Table 2:** Direct effect estimates of family income quintile on subjective well-being at adolescence, by childhood period, using marginal structural modeling.

Family income quintile	Early Childhood (ages 0–2)		Pre-School (ages 3–5)		Middle childhood (ages 6–8)		Model for Pre-adolescence (ages 9–11)	
	Estimate	95% CI	Estimate	95% CI	Estimate	95% CI	Estimate	95% CI
**Quintile 1 (poorest)**	-1.65	-2.91,-0.40	-0.21	-1.54,1.12	0.24	-1.07,1.54	-0.01	-1.38,1.36
**Quintile 2**	-0.63	-1.58,0.32	-0.02	-1.12,1.07	-0.08	-1.08,0.91	-0.45	-1.46,0.57
**Quintile 3**	-0.82	-1.74,0.10	-0.18	-1.16,0.79	0.16	-0.69,1.01	-0.23	-1.05,0.60
**Quintile 4**	-0.05	-0.77,0.68	-0.14	-0.94,0.67	0.43	-0.31,1.17	-0.42	-1.15,0.31
**Quintile 5 (richest)**	Reference		Reference		Reference		Reference	

Estimate from a marginal structural model using stabilized weights to account for time-invariant and time-varying covariates (sex, age, race/ethnicity, marital status, education, and work status of primary caregiver number of persons and of children in the family, birth year of child, state median income and state of residency) and the history of family income quintile throughout childhood (n = 1806). CI: confidence interval.

### Accumulation of risk model

Adolescents experienced a median of 3 years of low family income (two poorest family income quintiles) during their childhood. About a third (31%) of adolescents did not experience low family income during their childhood, 9% experienced 1 year of low family income, about 5% experienced 2 to 10 years of low family income, respectively, and 9% experienced low family income during their entire childhood (11 years).

Every additional year spent in low family income corresponded with a 0.10 (95% CI: 0.04, 0.16) lower SWB score in adolescence. This estimate was robust to sensitivity analyses and remained after adjusting for family income quintile and family socioeconomic variables during adolescence (S4 Table).

## Discussion

This study provides a life-course analysis of childhood family income on adolescent positive mental health. Results show that a lower family income in childhood related negatively to the SWB of adolescents, in line with previous cross-sectional evidence [23–26]. Low family income during childhood not only had a cumulative effect over time, but living in the poorest income quintile specifically during early childhood (age 0–2 years) had a lasting effect on adolescent SWB. Although no previous longitudinal study examined the effect of family income on youth positive mental health, studies on mental disorders support our findings [39]. A cumulative effect of low family income on poor adolescent mental health has been shown for depression [40, 41], anxiety [40], and antisocial behaviours [41]. Our study adds to this knowledge by showing that, in addition to mental problems, a low family income during childhood negatively relates to the development of positive mental health in youth.

The cumulative exposure to low family income during childhood was a significant and robust factor associated with poor adolescent SWB. Although the effect size for each year was small, the effect over childhood was cumulative. Given that low family income was defined as the two poorest quintiles, and thus representing about 40% of families, a cumulative exposure to low family income has the potential to impact a large portion of the population. The cumulative framework to socioeconomic inequalities has long been supported in adult research and suggests that cumulative economic hardship puts individuals at risk of experiencing continued economic, social and behavioral difficulties throughout their lives [42–44]. Our study contributes to the literature by showing a similar pattern of cumulative effect from childhood to adolescence, potentially affecting the life course even at this early life stage [4, 5]. These findings are compelling because reverse causality is less likely in children who usually do not have control over family income. From a materialistic perspective, children from poorer families may not have access to the same resources as other children. As a result, they may have greater difficulties in school, low self-efficacy, and low self-esteem [18]. From a psychosocial perspective, family economic deprivation is linked to greater exposure to adverse childhood experiences, family dysfunctions and harsher parental style [18]. The cumulative and compounded effect of these adversities may affect the brain [45] and dysregulate biological pathways [46], impacting the SWB of youths.

Early childhood was a sensitive period to low family income that was associated with a lasting disadvantage on adolescent SWB. Early childhood is a crucial time for social, emotional, and cognitive development and a low family income during this period may impede on processes relevant to SWB. Material deprivation during infancy is a strong risk factor for poor biological and social developmental outcomes [20, 28]. Financial hardship is also detrimental for the mental health of parents and may negatively affect parenting [18] with deleterious consequences for the development of the child [20]. Further, the first years of parenting may be an extremely stressful time for new parents which may be exacerbated by a low family income. Given that early childhood is often a time of reduced income for parents (e.g., due to parental work leave), the effect may be large at the population-level.

This study is the first to examine how family income during childhood is associated with positive mental health at adolescence, an understudied and formative stage of human development. The study is strengthened by careful use of MSM to account for endogenous confounding and several sensitivity analyses to check robustness of results. The PSID collected information on family income throughout the childhood of youths thereby reducing poor recall and allowing investigation of family income rather than a proxy measure for economic position. The study also has limitations. Although family income is an indicator of the childhood economic environment, it is not known if income was spent in a beneficial way for the child. Further, children from low income families may not necessarily experience economic disadvantage and vice versa due to how parents prioritize and spend money [47]. Many conditions lead families to low family income and we included the most common sociodemographic factors in our analysis; however unmeasured factors that correlate with income may still exist. Moreover, the study focused on the well-being of American adolescents in 2002 and 2007 and so the findings may not be generalizable to other countries or time periods.

In summary, low family income experienced in childhood related to positive mental health during adolescence. Results suggest that even a one-year reduction in experiencing low family income may be associated with significant benefits for the well-being of youth and that interventions that target the first few years of life may have the greatest impact. A small shift in adolescent positive mental health can potentially set the course to a lifetime of benefits at the population-level. There has been a call by public health agencies to protect and promote mental well-being in young individuals [48, 49]. Findings from this study point to the need for further research on the mechanisms that link low income to SWB that could inform public policies and intervention strategies.

## Supporting information

S1 Fig Diagram of the relationship between family income quintile and confounders over time. C represents time-invariant covariates, including age, sex and race/ethnicity of the primary caregiver, and birth year of the child. HIQ_1_, HIQ_2_, HIQ_3_, HIQ_4_, HIQ_5_, represent the family income quintile at childhood period 1 (early childhood), period 2 (pre-school years), period 3 (middle childhood), period 4 (pre-adolescence), and period 5 (adolescence), respectively. TVC_1_, TVC_2_, TVC_3_, TVC_4_, TVC_5_ represent time-varying covariates at each childhood period, respectively, including socioeconomic characteristics (marital status, education, work status) of the primary caregiver, number of persons and number of children living in the family. SWB_5_ denotes subjective well-being (SWB) measured at adolescence (period 5). Endogenous confounding exists because time-varying covariates (e.g. number of children in the family at TVC_2_) might be affected by prior family income quintile (e.g., HIQ_1_), but also confound the effect of later family income quintile (e.g., HIQ_3_) on the outcome (SWB_5_).
(DOCX)

S1 Table Items on the subjective well-being scale in the CDS-II and CDS-III. (DOCX)

S2 Table Characteristics of CDS participants measured at adolescence by missing data status. (DOCX)

S3 Table Sensitivity analyses of the direct effect estimates of family income quintile by childhood period on subjective well-being at adolescence, using marginal structural modeling. Estimates from a marginal structural model using stabilized weights to account for time-invariant and time-varying covariates (sex, age, race/ethnicity, marital status, education, and work status of primary caregiver number of persons and of children in the household, birth year of child, state median income and state of residency) and the history of household income quintile throughout childhood.
^*i*^ Multiple imputation by chained equations of missing data on time-varying marital status, education, and work status of the primary caregiver, number of persons and of children in the household at baseline. State of residency could not be imputed because of convergence problems and was not included in weight calculations.
(DOCX)

S4 Table Sensitivity analyses of the effect estimates of the number of years spent in the two poorest family income quintiles on subjective well-being at adolescence. Estimates from linear regression models, adjusted for time-invariant covariates (sex, age, race/ethnicity of primary caregiver, birth year of child) and time-varying covariates (marital status, education, and work status of the primary caregiver, number of persons and of children in the household, state median income and state of residency) at baseline.
^*i*^ Multiple imputation by chained equations of missing data on marital status, education, and work status of the primary caregiver, number of persons and of children in the household at baseline. State of residency could not be imputed because of convergence problems and was excluded from models.
(DOCX)

S1 File Details on sensitive period model specification. (DOCX)

## Abbreviations

CDS: Child Development Supplement
CI: Confidence interval
IPW: Inverse probability weights
MSM: Marginal structural modeling
PSID: Panel Study of Income Dynamics
USD: United States dollar
SD: Standard deviation
SWB: Subjective well-being
