# Supplementary material for: Early-life family income and subjective well-being in adolescents
Source: PLoS One. 2017 Jul 17;12(7):e0179380. doi: 10.1371/journal.pone.0179380 (PMC5513414; doi:10.1371/journal.pone.0179380)
Supplement: S1 Fig — C represents time-invariant covariates, including age, sex and race/ethnicity of the primary caregiver, and birth year of the child. HIQ1, HIQ2, HIQ3, HIQ4, HIQ5, represent the family income quintile at childhood period 1 (early childhood), period 2 (pre-school years), period 3 (middle childhood), period 4 (pre-adolescence), and period 5 (adolescence), respectively. TVC1, TVC2, TVC3, TVC4, TVC5 represent time-varying covariates at each childhood period, respectively, including socioeconomic characteristics (marital status, education, work status) of the primary caregiver, number of persons and number of children living in the family. SWB5 denotes subjective well-being (SWB) measured at adolescence (period 5). Endogenous confounding exists because time-varying covariates (e.g. number of children in the family at TVC2) might be affected by prior family income quintile (e.g., HIQ1), but also confound the effect of later family income quintile (e.g., HIQ3) on the outcome (SWB5). (DOCX) [file pone.0179380.s001.docx]

S1 Figure. Diagram of the relationship between household income quintile and confounders over time.

C represents time-invariant covariates, including age, sex and race/ethnicity of the primary caregiver, and birth year of the child. HIQ_1_, HIQ_2_, HIQ_3_, HIQ_4_, HIQ_5_, represent the household income quintile at childhood period 1 (early childhood), period 2 (pre-school years), period 3 (middle childhood), period 4 (pre-adolescence), and period 5 (adolescence), respectively. TVC_1_, TVC_2_, TVC_3_, TVC_4_, TVC_5_ represent time-varying covariates at each childhood period, respectively, including socioeconomic characteristics (marital status, education, work status) of the primary caregiver, number of persons and number of children living in the household. SWB_5_ denotes subjective well-being (SWB) measured at adolescence (period 5). Endogenous confounding exists because time-varying covariates (e.g. number of children in household at TVC_2_) might be affected by prior household income quintile (e.g., HIQ_1_), but also confound the effect of later household income quintile (e.g., HIQ_3_) on the outcome (SWB_5_).
