# Supplementary material for: Early-life family income and subjective well-being in adolescents
Source: PLoS One. 2017 Jul 17;12(7):e0179380. doi: 10.1371/journal.pone.0179380 (PMC5513414; doi:10.1371/journal.pone.0179380)
Supplement: S1 Table — (DOCX) [file pone.0179380.s002.docx]

#### S1 Table. Items on subjective well-being scale in the CDS-II and CDS-III by domains defined by Keyes [1].

| Domain | Items  “In the last month, how often did you feel…?” |
| --- | --- |
| Emotional well-being | 1. Happy |
|  | 1. Interested in life |
|  | 1. Satisfied |
| Social well-being | 1. That you had something important to contribute to society |
|  | 1. That you belonged to a community (like a social group, your school, or your neighborhood) |
|  | 1. That our society is becoming a better place for people like you |
|  | 1. That people are basically good |
|  | 1. That the way our society works made sense to you |
| Psychological well-being | 1. Good at managing the responsibilities of your daily life |
|  | 1. That you had warm and trusting relationships with others |
|  | 1. That you had experiences that challenged you to grow and become a better person |
|  | 1. Confident to think or express your own ideas and opinions |

The response scale for each item is

1 = never

2 = once or twice a week

3 = about once a week

4 = two or three times a week

5 = almost every day

6 = every day
