# Supplementary material for: Early-life family income and subjective well-being in adolescents
Source: PLoS One. 2017 Jul 17;12(7):e0179380. doi: 10.1371/journal.pone.0179380 (PMC5513414; doi:10.1371/journal.pone.0179380)
Supplement: S2 Table — (DOCX) [file pone.0179380.s003.docx]

S2 Table. Characteristics of CDS participants measured at adolescence by missing data status.

|  | Full data  n=1812 | Missing data at any childhood period  n=422 |
| --- | --- | --- |
|  | Weighted %(n)/Mean (SD) | Weighted %(n)/Mean (SD) |
| Characteristics of participants |  |  |
| Sex |  |  |
| Boys | 50.1 (894) | 52.9 (214) |
| Girls | 49.9 (898) | 47.1 (195) |
| Race/ethnicity |  |  |
| White | 74.9 (899) | 20.6 (194) |
| Black | 18.2 (817) | 11.2 (138) |
| Other | 6.8 (95) | 68.2 (190) |
| Part of sub-sample of immigrant families added in 1997-1999 |  |  |
| Yes | 0 (0) | 28.9 (230) |
| Characteristics of primary caregiver |  |  |
| Age (years) at year of birth | 29.2 (6.6) | 27.9 (5.8) |
| Sex |  |  |
| Women | 96.8 (1764) | 96.0 (395) |
| Men | 3.2 (48) | 4.0 (27) |
| Race/ethnicity |  |  |
| White | 78.8 (951) | 53.9 (186) |
| Black | 17.3 (804) | 12.6 (141) |
| Other | 3.9 (57) | 33.6 (95) |
| Marital status |  |  |
| Married/partnered | 70.1 (739) | 65.1 (181) |
| Not married/partnered | 29.9 (1069) | 34.9 (239) |
| Years of education | 13.6 (2.1) | 11.3 (3.8) |
| Working status |  |  |
| Full-time | 42.7 (846) | 40.1 (187) |
| Part-tine | 37.7 (610) | 40.7 (155) |
| Not working | 19.5 (352) | 19.3 (78) |
| Characteristics of household |  |  |
| Number of people in household | 3.9 (1.3) | 4.6 (1.7) |
| Number of children in household | 1.5 (1.2) | 2.0 (1.4) |
