# Supplementary material for: Early-life family income and subjective well-being in adolescents
Source: PLoS One. 2017 Jul 17;12(7):e0179380. doi: 10.1371/journal.pone.0179380 (PMC5513414; doi:10.1371/journal.pone.0179380)
Supplement: S3 Table — Estimates from a marginal structural model using stabilized weights to account for time-invariant and time-varying covariates (sex, age, race/ethnicity, marital status, education, and work status of primary caregiver number of persons and of children in the household, birth year of child, state median income and state of residency) and the history of household income quintile throughout childhood. i Multiple imputation by chained equations of missing data on time-varying marital status, education, and work status of the primary caregiver, number of persons and of children in the household at baseline. State of residency could not be imputed because of convergence problems and was not included in weight calculations. (DOCX) [file pone.0179380.s004.docx]

S3 Table. Sensitivity analyses of direct effect estimates of household income quintile by childhood period on subjective well-being at adolescence, using marginal structural modeling.

|  | Early Childhood  Ages 0-2 | Pre-  School  Ages 3-5 | Middle childhood  Ages 6-8 | Pre-adolescence  Ages 9-11 |
| --- | --- | --- | --- | --- |
|  | Estimate  [95% CI] | Estimate  [95% CI] | Estimate  [95% CI] | Estimate  [95% CI] |
| Restricted to subjective well-being measured in early adolescence (12-15 years) |  |  |  |  |
| Household income quintile |  |  |  |  |
| Quintile 1 (poorest) | -1.82 | -0.33 | -0.25 | 0.09 |
|  | [-3.23,-0.41] | [-1.82,1.17] | [-1.81,1.30] | [-1.46,1.63] |
| Quintile 2 | -0.39 | 0.10 | -0.51 | -0.05 |
|  | [-1.47,0.70] | [-1.13,1.33] | [-1.60,0.58] | [-1.24,1.15] |
| Quintile 3 | -0.63 | -0.28 | -0.07 | -0.02 |
|  | [-1.69,0.42] | [-1.37,0.80] | [-0.96,0.83] | [-0.98,0.94] |
| Quintile 4 | 0.16 | 0.05 | -0.10 | -0.18 |
|  | [-0.67,1.00] | [-0.86,0.97] | [-0.88,0.67] | [-1.02,0.67] |
| Quintile 5 (richest) | Reference | Reference | Reference | Reference |
|  |  |  |  |  |
| Restricted to subjective well-being measured in late adolescence (16-19 years old) |  |  |  |  |
| Household income quintile |  |  |  |  |
| Quintile 1 (poorest) | -1.36 | 1.28 | 0.19 | 0.10 |
|  | [-3.06,0.33] | [-0.96,3.52] | [-1.51,1.89] | [-1.72,1.92] |
| Quintile 2 | -0.76 | 0.22 | 0.36 | -1.00 |
|  | [-2.28,0.76] | [-1.50,1.93] | [-1.35,2.07] | [-2.25,0.25] |
| Quintile 3 | -0.82 | 0.16 | -0.09 | -0.34 |
|  | [-2.08,0.45] | [-1.31,1.63] | [-1.52,1.34] | [-1.47,0.78] |
| Quintile 4 | -0.05 | -0.54 | 1.46 | -0.85 |
|  | [-1.02,0.92] | [-1.55,0.47] | [0.18,2.75] | [-1.93,0.22] |
| Quintile 5 (richest) | Reference | Reference | Reference | Reference |
| Multiple imputation of missing values*^i^* |  |  |  |  |
| Household income quintile |  |  |  |  |
| Quintile 1 (poorest) | -1.21 | 0.20 | 0.44 | 0.11 |
|  | [-2.55,0.11] | [-1.17,1.57] | [-0.91,1.79] | [-1.24,1.47] |
| Quintile 2 | -0.25 | 0.36 | 0.03 | -0.30 |
|  | [-1.24,0.74] | [-0.81,1.53] | [-0.98,1.04] | [-1.35,0.76] |
| Quintile 3 | -0.40 | 0.07 | 0.42 | 0.07 |
|  | [-1.36,0.57] | [-0.98,1.12] | [-0.49,1.32] | [-0.80,0.94] |
| Quintile 4 | 0.45 | 0.26 | 0.67 | -0.15 |
|  | [-0.31,1.21] | [-0.59,1.11] | [-0.11,1.45] | [-0.89,0.59] |
| Quintile 5 (richest) | Reference | Reference | Reference | Reference |

Estimates from a marginal structural model using stabilized weights to account for time-invariant and time-varying covariates (sex, age, race/ethnicity, marital status, education, and work status of primary caregiver number of persons and of children in the household, birth year of child, state median income and state of residency) and the history of household income quintile throughout childhood.

***^I^*** Multiple imputation by chained equations of missing data on time-varying marital status, education, and work status of the primary caregiver, number of persons and of children in the household at baseline. State of residency could not be imputed because of convergence problems and was not included in weight calculations.
