# Supplementary material for: Early-life family income and subjective well-being in adolescents
Source: PLoS One. 2017 Jul 17;12(7):e0179380. doi: 10.1371/journal.pone.0179380 (PMC5513414; doi:10.1371/journal.pone.0179380)
Supplement: S4 Table — Estimates from linear regression models, adjusted for time-invariant covariates (sex, age, race/ethnicity of primary caregiver, birth year of child) and time-varying covariates (marital status, education, and work status of the primary caregiver, number of persons and of children in the household, state median income and state of residency) at baseline. i Multiple imputation by chained equations of missing data on marital status, education, and work status of the primary caregiver, number of persons and of children in the household at baseline. State of residency could not be imputed because of convergence problems and was excluded from models. (DOCX) [file pone.0179380.s005.docx]

S4 Table. Sensitivity analyses of effect estimates of number of years spent in two poorest household income quintile on subjective well-being at adolescence.

|  | Estimate  [95% CI] |
| --- | --- |
| Main analysis |  |
| Years in two lowest household income quintiles | -0.10 |
|  | [-0.16, -0.04] |
| Restricted to subjective well-being measured in early adolescence (12-15 years) |  |
| Years in two lowest household income quintiles | -0.10 |
|  | [-0.17, -0.03] |
| Restricted to subjective well-being measured in late adolescence (16-19 years) |  |
| Years in two lowest household income quintiles | -0.07 |
|  | [-0.16, 0.02] |
| Restricting to childhood period between ages 3-11 |  |
| Years in two lowest household income quintiles | -0.11 |
|  | [-0.18, -0.04] |
| Restricting to those in the two poorest income quintiles for ≤ 5 years |  |
| Years in two lowest household income quintiles | -0.15 |
|  | [-0.28, -0.02] |
| Adjusted for household income quintile at birth year |  |
| Years in two lowest household income quintiles | -0.09 |
|  | [-0.17, -0.02] |
| Adjusted for household income quintile at adolescence |  |
| Years in two lowest household income quintiles | -0.09 |
|  | [-0.16, -0.02] |
| Adjusted for all family socioeconomic variables at adolescence |  |
| Years in two lowest household income quintiles | -0.09 |
|  | [-0.15, -0.02] |
| Lowest household income quintile |  |
| Years in lowest household income quintile | -0.07 |
|  | [-0.18, 0.03] |
| Multiple imputation of missing values*^i^* |  |
| Years in lowest household income quintile | -0.10 |
|  | [-0.16, -0.04] |

Estimates from linear regression models, adjusted for time-invariant covariates (sex, age, race/ethnicity of primary caregiver, birth year of child) and time-varying covariates (marital status, education, and work status of the primary caregiver, number of persons and of children in the household, state median income and state of residency) at baseline.

***^I^*** Multiple imputation by chained equations of missing data on marital status, education, and work status of the primary caregiver, number of persons and of children in the household at baseline. State of residency could not be imputed because of convergence problems and was excluded from models.
