# Supplementary material for: Early-life family income and subjective well-being in adolescents
Source: PLoS One. 2017 Jul 17;12(7):e0179380. doi: 10.1371/journal.pone.0179380 (PMC5513414; doi:10.1371/journal.pone.0179380)
Supplement: S1 File — (DOCX) [file pone.0179380.s006.docx]

S1 File. Details on sensitive period model specification.

The diagram for the relationship between the study variables over time is illustrated in Appendix 2. First, we estimated the total effect of household income quintile (${HIQ}_{ij}$) on adolescent subjective well-being score (${SWB}_{i}$) by fitting a linear regression separately for each childhood period (*j*) (childhood period 1 = early childhood; period 2 = pre-school years; period 3 = middle childhood; and period 4 = pre-adolescence). We adjusted for vectors of time-invariant confounders ($C_{i}$) (age, sex, race/ethnicity of the primary caregiver, and birth year of the child); time-varying covariates at baseline (${TVC}_{i1}$) and at the preceding childhood period of interest (${TVC}_{ij-1}$) (household income quintile, number of persons and of children in the household; marital status, education, and work status of the primary caregiver); and characteristics of state of residency during the childhood period of interest (*SC_ij_*) (state median income and state of residency):

$$E\left[ {SWB}_{i} | {HIQ}_{ij}=hiq, {C_{i}= c, TVC}_{ij}= tvc, {SC}_{ij}={sc}_{ij} \right]=\beta_{0}+ \beta_{1}{hiq}_{ij}+\beta_{2}c_{i}+\beta_{3}{tvc}_{i1}+\beta_{4}{tvc}_{ij-1}+{sc}_{ij}$$

The coefficient β_1_ provides estimation of the total effect of household income quintile of the specified childhood period, assuming no unmeasured confounding. The total effect encompasses both the direct effect and indirect effect that may be mediated through household income quintiles at later childhood periods or adolescence.

Second, we estimated the controlled direct effect of household income quintile of each childhood period (${HIQ}_{ij}$) using marginal structural modelling (MSM). The objective was to test whether there was a sensitive period in childhood during which household income quintile had a lasting direct effect on adolescent SWB that was not explained through household income quintiles later in childhood or adolescence, after accounting for time-invariant and time-varying confounders. The estimation of the controlled direct effect assumes no unmeasured confounding between household income quintiles across childhood periods and with adolescent SWB. We fitted a linear model in the following form:

$$E\left[ {SWB}_{i} | {HIQ}_{ij}=hiq \right]=\alpha_{0}+ \alpha_{1}{hiq}_{i1}+ \alpha_{2}{hiq}_{i2}+ \alpha_{3}{hiq}_{i3}+ \alpha_{4}{hiq}_{i4}+ \alpha_{5}{hiq}_{i5}$$

where $\alpha_{i1}$, $\alpha_{i2}$, $\alpha_{i3}$, $\alpha_{i4}$, $\alpha_{i5}$ represent the estimation of the direct effect of household income quintile (*hiq*) in period *j* (period 1 = early childhood; period 2 = pre-school years; period 3 = middle childhood; period 4 = pre-adolescence; period 5 = adolescence). We found no evidence of interaction between household income quintiles at different time points and therefore did not include any interaction terms in the model. MSM accounts for time-varying variables throughout childhood by weighting the sample using inverse probability weights (IPW). MSM assumes no unmeasured confounding, positivity, and correct model specification [2]. The weights are calculated by estimating the probability of a child of having the household income quintile at childhood period *j* that he or she in fact had, conditional on past covariates and history of household income quintile. We calculated stabilized weights for each time period *j*:

$$W_{i}^{j}= \frac{P\left( {HIQ}_{ij}={hiq}_{ij} \right|{HIQ}_{i1}={hiq}_{i1}, \ldots,{HIQ}_{ij-1}={hiq}_{ij-1})}{P\left( HIQ={hiq}_{ij} \right|C=c_{i}, {TVC}_{i1}={tvc}_{i1},\ldots, {TVC}_{ij-1}={tvc}_{ij-1}, {HIQ}_{i1}={hiq}_{i1}, \ldots,{HIQ}_{ij-1}={hiq}_{ij-1})}$$

We calculated the numerator of the weight by regressing household income quintile (*HIQ*) at the childhood period of interest *j* on the history of HIQ up to that point (*HIQ_i_*_1_ *to HIQ_ij-_*_1_) in an ordinal logistic model. We calculated the denominator by regressing household income quintile (*HIQ*) at the childhood period of interest on time-invariant covariates (*C*), the history of time-varying covariates (TVC*_i_*_1_ *to TVC_ij-_*_1_) and the history of household income quintiles (*HIQ_i_*_1_ *to HIQ_1j-_*_1_) up to that point in an ordinal logistic model. To ensure correct model specification [2], we checked that the final mean weight was close to 1, with a small standard deviation (SD) and narrow range (mean: 0.92, SD: 0.82, range: 0.12-5.41). The final weight was the product of the calculated weights across all time periods and the survey weight:

$${final w_{i}=w}_{i}^{1}*w_{i}^{2}* w_{i}^{3}* w_{i}^{4}* w_{i}^{5}* w_{i}^{s}$$

# References for Appendix

1 Keyes CL. Mental health in adolescence: is America's youth flourishing? *The American journal of orthopsychiatry* 2006;**76**:395-402.

2 Cole SR, Hernán MA. Constructing inverse probability weights for marginal structural models. *American Journal of Epidemiology* 2008;**168**:656-64.
